# Influence of Bacterial Biofilm Polysaccharide Structure on Interactions with Antimicrobial Peptides: A Study on *Klebsiella pneumoniae*

**DOI:** 10.3390/ijms19061685

**Published:** 2018-06-06

**Authors:** Barbara Bellich, Cristina Lagatolla, Alessandro Tossi, Monica Benincasa, Paola Cescutti, Roberto Rizzo

**Affiliations:** Department of Life Sciences, University of Trieste, Via Licio Giorgieri 1, 34127 Trieste, Italy; bbellich@units.it (B.B.); clagatolla@units.it (C.L.); atossi@units.it (A.T.); mbenincasa@units.it (M.B.); pcescutti@units.it (P.C.)

**Keywords:** bacterial biofilm, matrix exopolysaccharides, antimicrobial peptides, bacterial protection, exopolysaccharides biological activity

## Abstract

Biofilms are complex systems produced by bacteria and constituted by macromolecular matrix embedding cells. They provide advantages to bacteria including protection against antimicrobials. The protection given by biofilms produced by *Klebsiella pneumoniae* strains towards antimicrobial peptides of the innate immune system was investigated. In particular, the role of matrix bacterial exopolysaccharides was explored. Three clinical strains producing exopolysaccharides with different chemistry were selected and the interaction of purified biofilm polysaccharides with two bovine cathelicidins was studied by circular dichroism spectroscopy and microbiological assays to establish their influence on the peptide’s antimicrobial activity. The spectroscopic data indicated a different extent of interaction with the two peptides, in a manner dependent on their sugar composition, and in particular the presence of rhamnose residues correlated with a lower interaction. The extent of interaction was then related to the protection towards antimicrobial peptides, conferred by the addition of the different exopolysaccharides, in minimum inhibitory concentration (MIC) assays against a reference *Escherichia coli* strain. Microbiological results were in very good agreement with spectroscopic data, confirming the active role of matrix polysaccharides in determining a biofilm’s protective capacity and indicating lower protection levels afforded by rhamnose containing exopolysaccharides.

## 1. Introduction

Biofilms are the most common way of life for bacteria [1]. A main characteristic of these complex communities is the production of a gelling matrix [2,3] which includes the bacterial cells and protects them against external threats, whether mechanical or chemical. Most biofilms are produced after adhesion of bacterial cells to a surface, but floating pellicles have also been described in the literature [4]. Of particular relevance for human health is biofilm production on internal tissues or medical devices, which is an important cause of chronic infections, often difficult to eradicate. Besides intrinsic bacterial resistance to antimicrobial agents and antibiotics of sessile microbes, biofilms may also enhance the resistance level because of the increased difficulty of drug molecules permeating inside the matrix: in fact, the permeation kinetics are impaired by the gel-like structure of the matrix, while specific interactions with matrix components may lead to the sequestering of antimicrobials [5,6].

Biofilm matrices are complex entities, composed of different macromolecules, including polysaccharides, proteins and extracellular DNA, as well as of molecular species with lower molecular masses such as lipids. Polysaccharides are considered to be mainly responsible for setting up of the matrix architecture, as they can establish intermolecular interactions with themselves and with other macromolecules, such as proteins [7,8]. These interactions confer stability to the macromolecular scaffold where bacterial cells are hosted. The matrix can be modelled as a sort of macromolecular network, swollen by water, comprising channels through which small molecules can circulate to reach bacteria and interact with their membrane. This means that the small molecules encounter polysaccharides on their way and can interact with them to different extents. Although this rather coarse picture of the biofilm architecture is generally accepted, the details of the structure and interactions of its components are not well known. In particular, also considering the great variety of their chemical structures, the specific role of matrix polysaccharides is mostly unknown.

Infected organisms combat bacterial infections using several different weapons, among which a relevant and interesting role is played by the antimicrobial peptides (AMPs) of the innate immune system [9]. In many organisms these constitute a first line of defence against bacteria and they are being actively investigated as possible alternatives to conventional antibiotics that may help solve the burgeoning problem of antibiotic resistance. It is therefore interesting to investigate the possible interactions between AMPs and polysaccharide constituents of biofilm matrices and to determine if they help bacteria included in biofilms to escape the action of these relevant innate immune effectors. It is obvious that if interactions do occur, they depend on structural features of both polysaccharides and peptides, so structural definition is important. In fact, the chemistry of biofilm polysaccharides depends not only on the producing bacterial strains but also on environmental conditions, so that bacteria can conveniently modulate it in order to respond efficiently to external stresses, including the action of antimicrobials.

In a previous investigation carried out on exopolysaccharides produced by *Pseudomonas aeruginosa*, *Inquilinus limosus*, and two species of the *Burkholderia cepacia* complex [10], we were able to show that the biopolymers obtained under planktonic conditions were able to interact with cathelicidin antimicrobial peptide of different mammalian origin. In particular, the human cathelicidin LL-37, upon interaction with the exopolysaccharides, adopts a α-helical conformation and becomes bound to the polysaccharide backbone, thus impairing its action on the bacterial membranes of matrix embedded bacteria [11,12].

*Klebsiella pneumoniae* is an enteric gram-negative bacillus causing a wide range of hospital-acquired infections, among which ventilator-associated pneumonia and urinary tract infections are the most frequent, especially in patients who undergo procedures with inserted medical devices such as endotracheal tubes or catheters. This is due to the ability of *Klebsiella* to adhere both to biotic and abiotic surfaces thanks to its sticky phenotype conferred by production of fimbriae and exopolysaccharides, which favour biofilm formation [13].

In this study, three clinical strains of *Klebsiella pnemoniae* (named KpTs101, KpTs113, and KpMn7), collected from urinary tract infections and producing three different polysaccharides (extracellular polysaccharides (EPOLs)), have been investigated [14,15,16]. The structures of these polysaccharides, defined in our laboratory, are very different to each other and are described in Table 1. The structure of the polysaccharide produced by the strain KpMn7 is identical to that published by Kubler-Kielb et al. [16].

The exopolysaccharides were purified from biofilms (BF) and investigated in the presence of two bovine cathelicidins: BMAP-27 and Bac7(1–35). The former is a membranolytic helical peptide, the latter is a fully active fragment of Bac7, which translocates into susceptible bacteria by using a specific transporter, where it then interferes with ribosomes [17].

Strain KpTs101 produces two similar polysaccharides that are identical to the O-chains of the lipopolysaccharides characterized from *K. pneumoniae* serotype O1. Of particular interest is the fact that the exopolysaccharide present in biofilm from the strain KpMn7 is rich in rhamnose residues. This is a 6-deoxy sugar having methyl groups on the C-6 position, in place of the more usual primary alcohol function. Compared with more common aldohexoses, this confers a distinctly less polar character to the polymer, which might favour hydrophobic interactions within itself or with other components of the biofilm matrix.

The interactions between polysaccharides and antimicrobial peptides were investigated using circular dichroism spectroscopy in systems composed of the purified exopolysaccharide and peptides and by microbiological assays, in which a bacterial strain was exposed to the antimicrobial peptides in the presence of exopolysaccharides. This test was performed using the reference strain *E. coli* ML-35 rather than a *Klebsiella* one to get rid of the interference of exopolysaccharides produced by bacteria themselves during the test.

## 2. Results and Discussion

BMAP-27 and Bac7(1–35) have a different mechanism of action towards bacteria. Upon interaction with bacterial membranes, BMAP-27 acquires an amphipathic α-helical conformation, with the polar sector of the cylinder characterized by the presence of numerous positively charged residues, and the opposite one having a markedly hydrophobic character. This configuration favours interaction with, and insertion into, the bacterial membrane, and the peptides then aggregate to form pores and/or disaggregates the membranes by the so-called “carpet mechanism” in a concentration dependent manner [18]. On the contrary, Bac7(1–35), after crossing the membrane without lysis, acts by targeting ribosomes [17]. Because of these very different modes of actions, the following description will consider the two peptides separately.

### 2.1. Interaction of Biofilms EPOLs and BMAP-27 AMP

**Figure 1:**
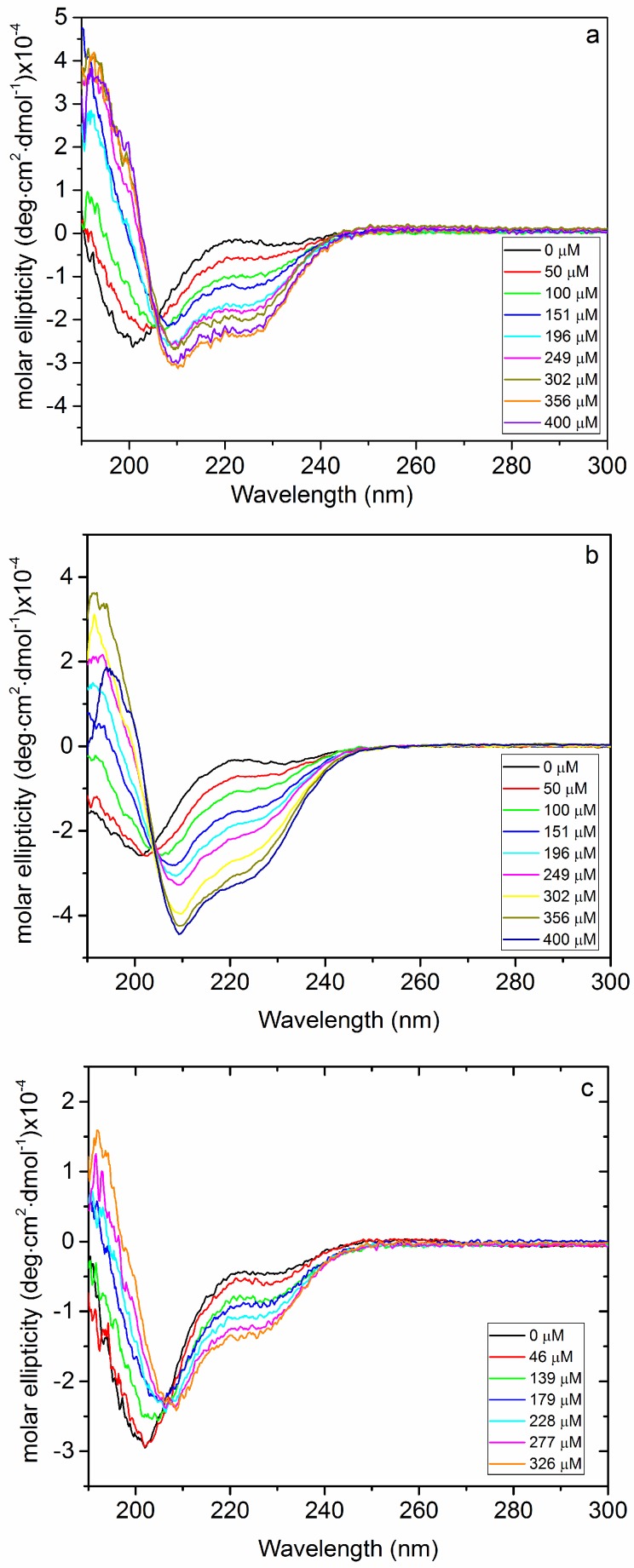
Circular dichroism (CD) spectra of BMAP-27 as a function of increasing extracellular polysaccharide (EPOL) concentrations. (**a**) KpTs101; (**b**) KpTs113; (**c**) KpMn7. Polysaccharide concentrations are indicated. [BMAP-27] = 20 μM, T = 25 °C, phosphate buffer.

**Figure 2:**
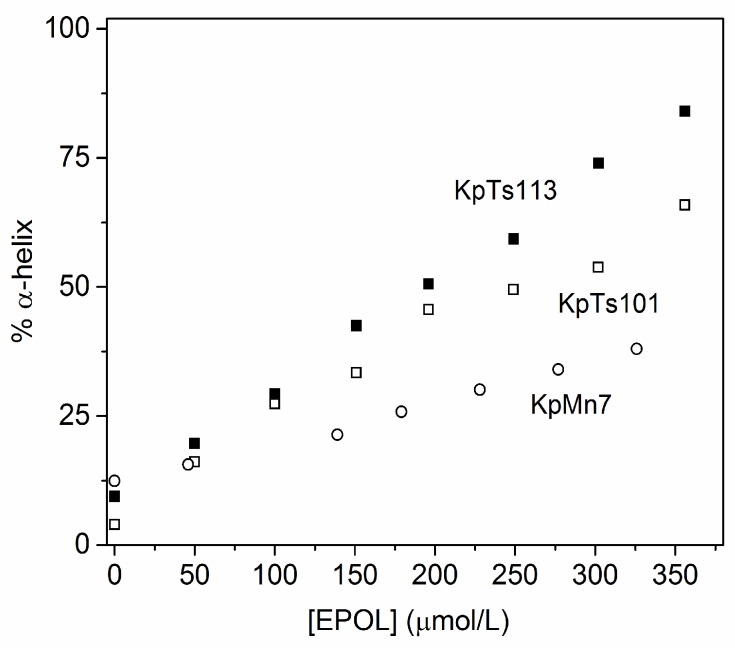
Percentage of α-helix of BMAP-27 induced by the EPOLs purified from KpTs101, KpTs113, and KpMn7 biofilms. [BMAP-27] = 20 μM, T = 25 °C, phosphate buffer.

Circular dichroism experiments were carried out on solutions with a fixed peptide concentration (20 μM) and increasing EPOLs concentrations. They showed that BMAP-27 is induced to adopt a α-helical conformation in the presence of EPOLs from BF produced by all three strains, KpTs101, KpTs113, and KpMn7, but to different extents (Figure 1). From the spectra in Figure 1 it is clear that, while KpTs101 and KpTs113 EPOLs have rather similar helix-inducing effects, KpMn7 EPOL is less effective in inducing the conformational change. This result can be quantified and better visualized by transforming molar ellipticity values into percentage α-helix content, induced by the three polysaccharides (Figure 2). 

**Figure 3:**
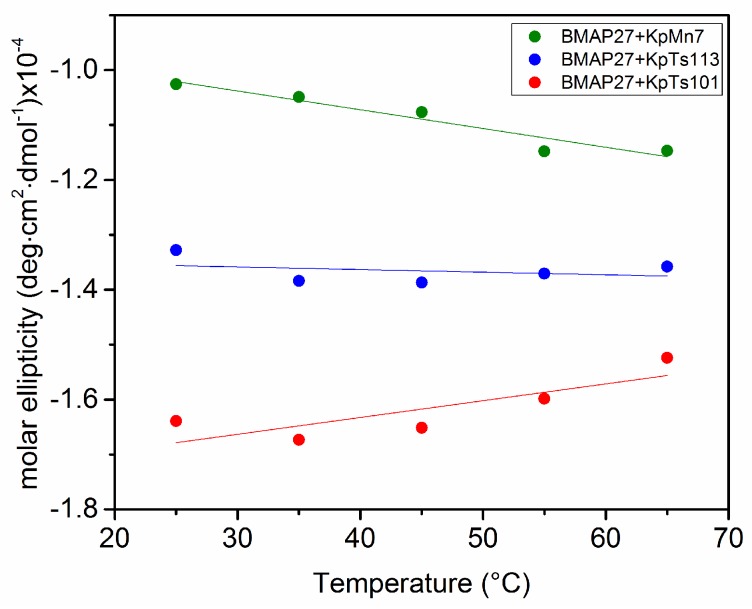
Increasing temperature effect on the BMAP-27 conformation in phosphate buffer in the presence of the investigated EPOLs. The CD ellipticity was measured at 222 nm. The EPOL concentrations were KpTs101 = 200 µM; KpTs113 = 198 µM; KpMn7 = 179 µM.

**Figure 4:**
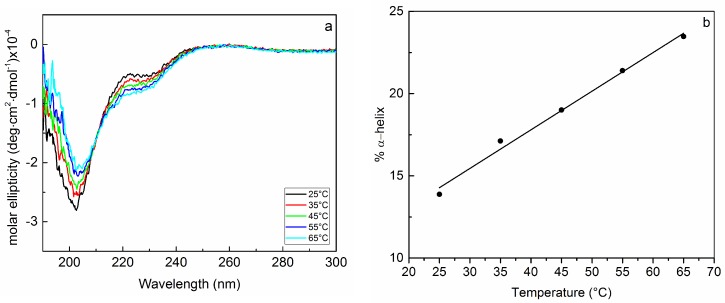
(**a**) Temperature induced CD spectra modifications for BMAP-27 in phosphate buffer; (**b**) Percent of α-helix content in BMAP27 phosphate buffer solutions as a function of the temperature.

Both KpTs101 and KpTs113 EPOLs are able to induce a considerable level of α-helical conformation (as compared also to that induced by 50% trifluoroethanol [19]), with KpTs113 being the most efficient. On the contrary, the KpMn7 EPOL induces only 25% of ordered conformation at the same concentration. The data clearly show that the interaction of KpMn7 EPOL with BMAP-27 is less intense than that given by the other polysaccharides. The stability of the interaction between EPOLs and BMAP-27 was also investigated as a function of the temperature (Figure 3) with the EPOL concentration set in the middle of the range used in the above described experiments (See legend of Figure 4).

In good agreement with the data of Figure 2, the system containing EPOL KpTs113, which interacted rather strongly with the peptide, did not show any variation in circular dichroism (CD) spectra upon increasing temperature; EPOL KpTs101, which induced the highest content of α-helical conformation in BMAP-27, showed a small decrease of the ordered conformation upon increasing the temperature (positive slope of the curve in Figure 3) probably due to small dissociation of the α-helix-stabilized complex. Rather surprisingly, EPOL KpMn7 exhibited a negative slope indicating an increase of the negative α-helical band at 222 nm.

Since, among the investigated EPOLs, KpMn7 exhibited the lowest degree of interaction with BMAP-27, suggesting a rather high concentration of free peptide in solution, the temperature behaviour of the peptide alone in phosphate buffer was also investigated. This experiment showed a 10% increase of the α-helix content driven by the temperature increase (Figure 4). This behaviour was almost identical to that reported in Figure 3 and obtained for the exopolysaccharide KpMn7, further supporting the low extent of interactions between the peptide and this BF component. To explain the increase of the peptide α-helix content with temperature, it might be speculated that by raising the energy of the system, intermolecular interactions with solvent are disrupted in favour of the intramolecular ones characterized by the α-helical hydrogen bonds. The temperature behaviour of the peptide alone nicely explains why the system containing EPOL KpMn7 and BMAP-27 exhibited a small increase in the peptide ordered conformation content as a function of the temperature. The rather weak interaction between EPOL KpMn7 and BMAP-27 indeed leaves a not minor amount of free peptide, which might increase by increasing the temperature; consequently, the temperature behaviour of the BMAP-27/EPOL KpMn7 system is dominated by the behaviour of the free peptide.

The interaction between BMAP-27 and KpTs101, KpTs113, and KpMn7 EPOLs, and its relation with bacterial protection, was then confirmed by evaluating the EPOLs’ ability to reduce the antimicrobial activity of the AMP against Escherichia coli ML-35 planktonic cells using the minimum inhibitory concentration (MIC) assay. Preliminary experiments showed that the three *Klebsiella* isolates were more resistant than *E. coli* ML-35 to both AMPs (Table 2) probably due to the action of the EPOLs produced by these strains.

For this reason, a different bacterium was selected for MIC measurements in the presence of EPOLs extracted from *K. pneumoniae* BF, as indicated in the introduction.

**Figure 5:**
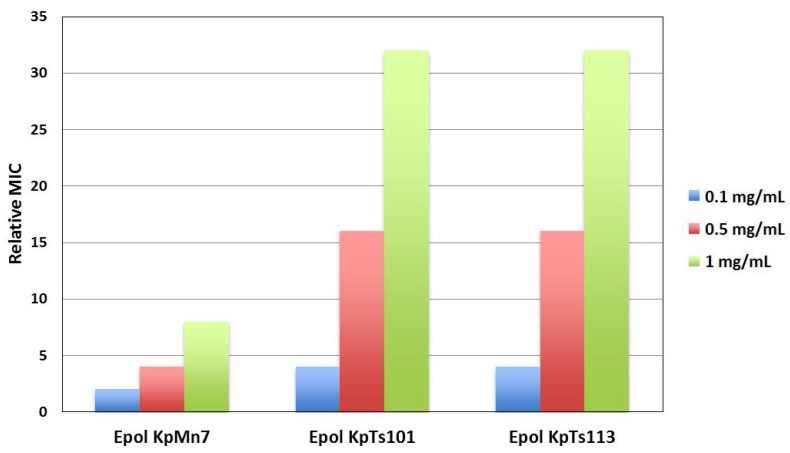
Relative MIC values for BMAP-27 against *Escherichia coli* ML-35 in the presence of different concentrations of *K. pneumoniae* EPOLs. The relative MIC is the ratio between the MIC value in the presence and in the absence of the polysaccharide. The MIC value in the absence of polysaccharides was 2 µM. The values were obtained from three independent experiments.

The MIC experiments (Figure 5) fully confirmed the spectroscopic findings, revealing a lower protective effect of KpMn7 EPOL compared to the other two EPOLs.

### 2.2. Interaction of Biofilms EPOLs and Bac7(1–35) AMP

**Figure 6:**
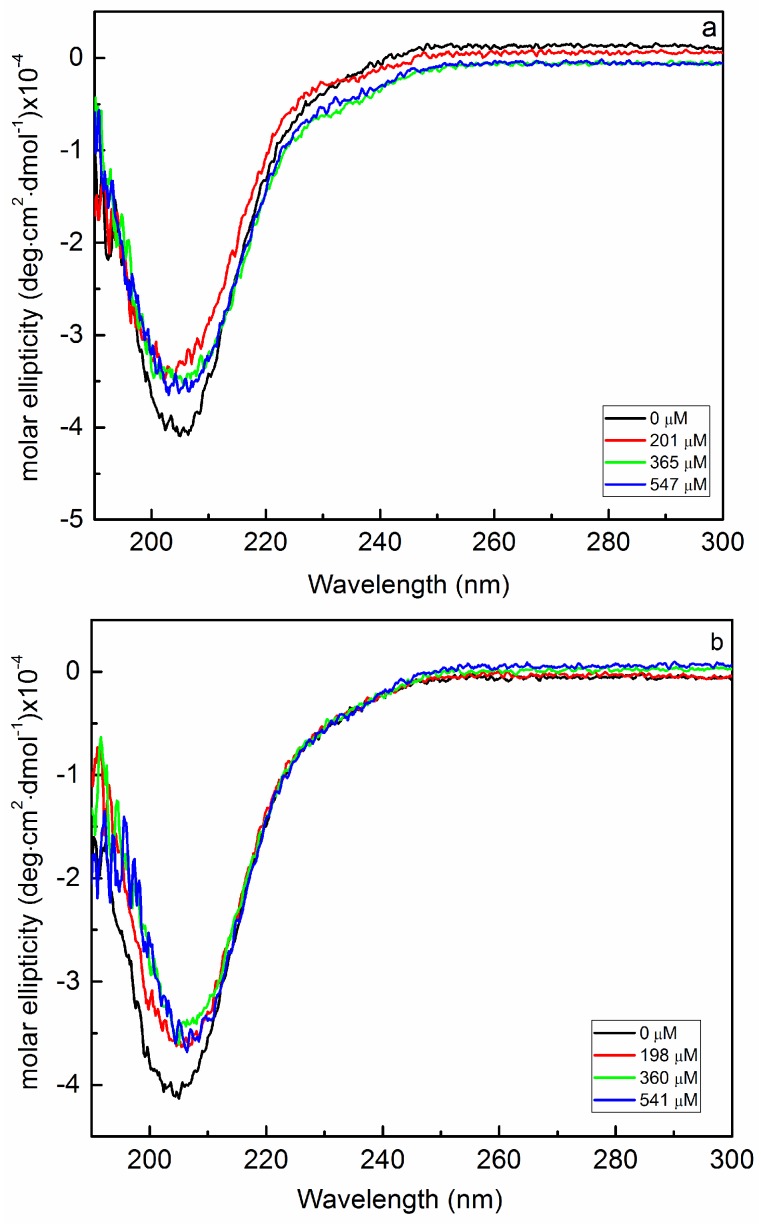

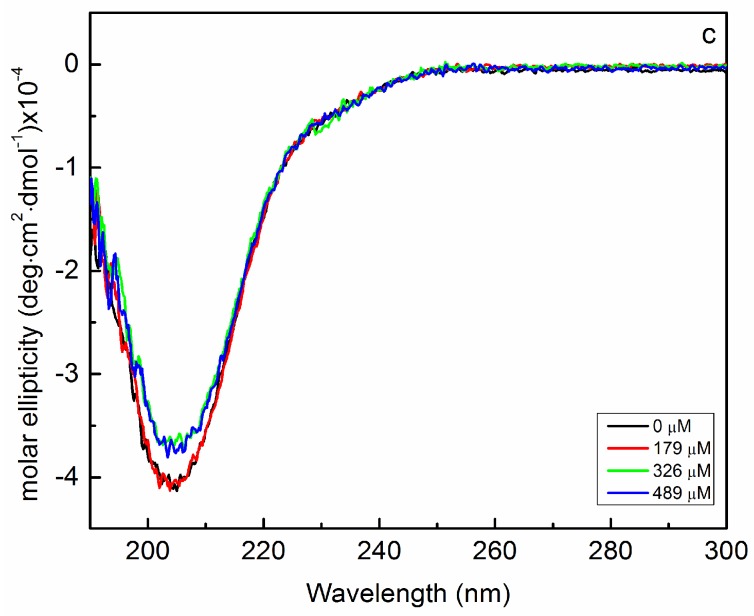
Bac7(1–35) CD changes in phosphate buffer as a function of increasing concentration of polysaccharides extracted from KpTs101 (**a**); KpTs113 (**b**); and KpMn7 (**c**) biofilms.

Bac7(1–35), a proline-rich antimicrobial peptide, adopts a conformation quite different from the α-helical one of BMAP-27. In fact, Bac7(1–35) CD spectrum in phosphate buffer recalls that of the polyproline II conformation (Figure 6a, black curve) [20], which is not surprising considering the high percentage of proline residues in its primary structure. The addition of *K. pneumoniae* EPOLs to a Bac7(1–35) buffered solution caused small variations in the CD spectra (Figure 6) indicating some change in the peptide conformation.

The small changes observed might be due either to limited interaction between the peptide and the EPOLs or to the fact that interaction do exists but does not greatly affect the peptide conformation. Nevertheless, the lowest spectroscopic variation in the CD curves was obtained for the peptide/KpMn7 EPOL system, as in the case of the BMAP-27 study. In fact, the first addition of the polysaccharide (Figure 6c, red curve) did not change the shape of the spectrum, which is identical to that of the peptide alone.

**Figure 7:**
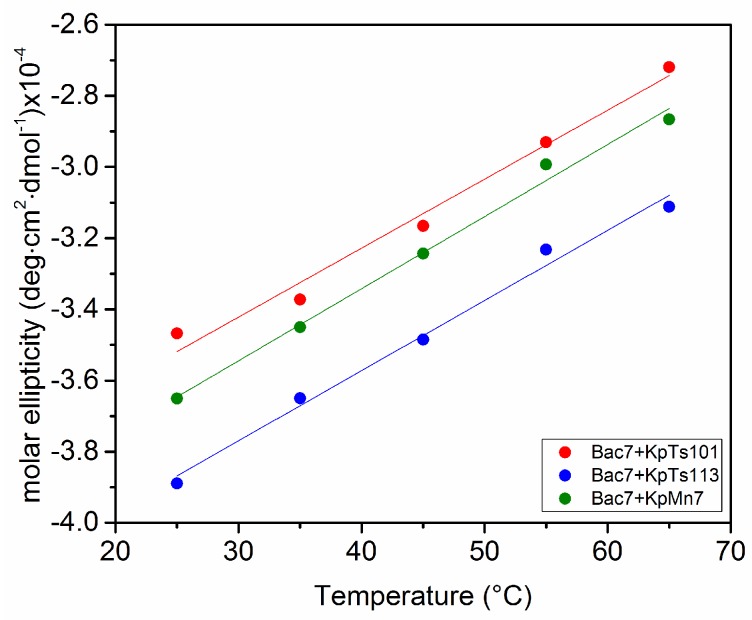
Increasing temperature effect on the Bac7(1–35) conformation in phosphate buffer in the presence of the investigated EPOLs. The CD ellipticity was measured at 205 nm. The EPOL concentrations were KpTs101 = 201 μM; KpTs113 = 198 μM; KpMn7 = 489 μM.

In order to better characterize the possible interactions, the Bac7(1–35)/EPOLs systems were also investigated as a function of the temperature in the range 25 to 65 °C (Figure 7).

Figure 7 shows that, upon increasing the temperature, the polyproline II type spectra changed for all the investigated systems with an almost identical slope. The only observation which might be derived is that Bac7(1–35) in the presence of EPOL KpTs113 exhibited a lower CD intensity in the temperature range explored.

**Figure 8:**
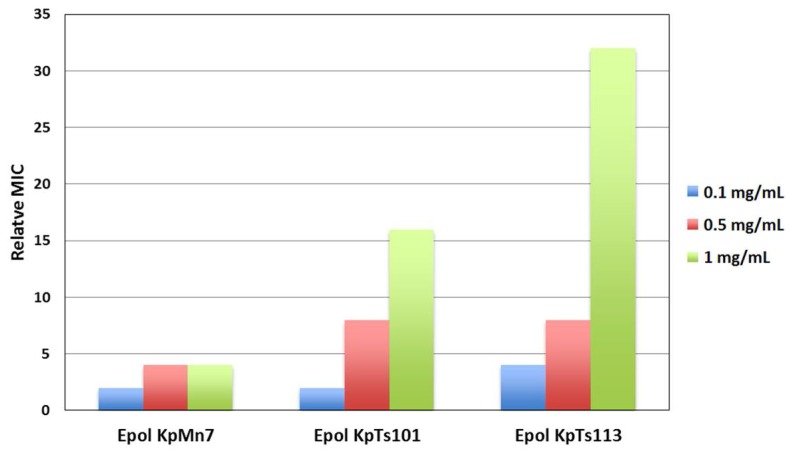
Relative MIC values for Bac7(1–35) against *Escherichia coli* ML-35 in the presence of different concentrations of *K. pneumoniae* EPOLs. The relative MIC is the ratio between the MIC value in the presence and in the absence of the polysaccharide. The MIC value in the absence of polysaccharides was 2 µM. The values were obtained from three independent experiments.

As for the BMAP-27 containing systems, the evaluation of Bac7(1–35) activity against *E. coli* ML-35 by MIC assays in the presence of different concentrations of the investigated EPOLs (Figure 8) was carried out and the results confirmed the trend suggested by the spectroscopic data. EPOL from KpMn7 biofilms did not significantly increase MIC values, indicating a protection lower than that offered by the other two EPOLs. KpTs113 EPOL was the most effective, while KpTs101 exhibited an intermediate activity. Thus, the extent of protection followed the trend KpTs113 > KpTs101 > KpMn7, in a dose-dependent manner, for the first two polysaccharides.

## 3. Materials and Methods

### 3.1. Bacterial Strains and Biofilm Culture

Three clinical isolates of *Klebsiella pneumoniae,* were collected from patients with urinary tract infections attending hospitals of the Friuli Venezia Giulia region (Italy) and named KpTs101, KpTs113, and KpMn7. For the production of biofilms, *K. pneumoniae* strains were grown on cellulose membranes deposited on solid agar medium [21]. Cellulose membranes (Sigma, St. Louis, MO, USA, cut-off 14,000 Da) were cut in circle the size of the Petri dish (90 mm Ø), washed first in a boiling solution of 5% Na_2_CO_3_ for 15 min and then in boiling water for 15 min, autoclaved and placed over Petri dishes, containing Müller Hinton (MH) solid medium. The excess of water was removed before seeding the bacteria. Two drops of 10 µL each of an overnight liquid culture of *K. pneumoniae* strain in MH broth were placed on the cellulose membranes. After 2 days of incubation at 30 °C, the material from each Petri dish was scraped from the membranes using about 3 mL of 0.9% NaCl, centrifuged at 48,000× *g* at 4 °C for 20 min, and filter-sterilized (Millipore membranes 0.22 μm, Merck KGaA, Darmstadt, Germany). UV spectroscopy was used to check for the presence of proteins, which eventually were eliminated by incubating the solution with protease (from *Streptomyces griseus*, Sigma) at 37 °C for 16 h, followed by centrifugation to remove insoluble material. EPOLs were precipitated from the solutions with 4 volumes of cold ethanol, centrifuged, dissolved in water and dialyzed (cut-off 14,000 MM) first against 0.1 M NaCl, then water, taken to pH 6.5–7.0, filtered on membranes (Millipore, cut-off 0.45 µm) and recovered by lyophilisation.

### 3.2. Antimicrobial Susceptibility Testing

Peptides were purchased from NovoPro Bioscience Inc. (Shanghai, China). The purity of peptides was ≥90%, and the molecular weight was verified by ESI mass spectrometry. Antimicrobial susceptibility testing was carried out by the microdilution susceptibility assay according to the CLSI guidelines [22]. Briefly, 5 × 10^5^ bacterial cells/mL were inoculated in presence of twofold dilutions of each AMP and incubated for 18 h at 37 °C. The MIC (minimum inhibitory concentration) was the lowest concentration of AMP that completely inhibited growth of bacteria. To evaluate the protective effect of the different EPOLs, MIC value of each peptide was evaluated against *Escherichia coli* ML-35 (that does not produce polysaccharides similar to those of *Klebsiella*) both in the absence and in presence of different concentrations of the EPOLS purified from biofilm produced by KpTs101, KpTs113, and KpMn7. The experiments were repeated at least three times in duplicate.

### 3.3. Circular Dichroism Spectroscopy

All CD measurements were performed on a Jasco J-710 instrument (JASCO Inc., Easton, MD, USA) either at 25 °C or as a function of the temperature (from 25 to 65 °C, step of 10 °C). Spectra were recorded in 10 mM sodium phosphate buffer at pH 7.4, using 2 mm quartz cells in the wavelength region from 190 to 300 nm. A correction for solvent baseline and polysaccharide contribution was made digitally in each case. Analyses were performed using a peptide concentration of 20 µM and varying the polysaccharide concentrations (referred to the mass of the polysaccharide’s repeating unit) as indicated in figures. Spectra were the result of accumulation of two scans. In all experiments, the polysaccharide-buffered solution was added to the buffered solution of peptide up to the desired concentration.

The percentage of α-helix content was determined as [θ]/[θ]_α_, where [θ] is the observed molar ellipticity at 222 nm and [θ]_α_ the molar ellipticity of a fully structured peptide calculated using the equation [θ]_α_ = −40,000·(1 − 2.5/*n*), where *n* is the number of amino acid residues in the peptide and −40,000 the estimated ellipticity of a fully structured infinitely long helix [23,24].

## 4. Conclusions

Polysaccharides have an important and probably unique role in biofilm matrices, and it is becoming increasingly clear that this role is not limited to simply being a physical constituent of the porous scaffold entrapping bacterial cells and allowing permeation of active small molecules. In addition, their protective effect is not limited to that of a physical barrier blocking or limiting the penetration dynamics of molecular species dangerous for bacterial colonies. The high variability of BF polysaccharide chemical structures and the finding that a single bacterial species can produce many different polysaccharides [25] suggest that these polymers can play specific biological roles affecting the functioning of the biofilm way of bacterial life.

In a previous investigation on the interaction of AMPs with exopolysaccharides produced by bacteria different from those considered in this paper, we proposed a model [10] of peptide–polysaccharide backbone interaction where the peptide adopts an ordered conformation. In this way, the more hydrophilic sector of the peptide secondary structure can interact with the hydroxyl groups of the polysaccharide backbone throughout polar contacts. The formation of these complexes, detected by the increase of the AMP ordered conformation, disfavors the interaction of AMPs with the bacterial membranes and eventually their antimicrobial action.

In this study, we investigated a possible role of exopolysaccharides extracted from biofilms produced by three clinical strains of *Klebsiella pneumoniae*. They exhibit different structures so that the relation between monosaccharide composition and activity could be explored. Specifically, KpTs113 EPOL has an ionic character due to the presence of an uronic acid, while KpTs101 EPOL is composed of two neutral galactans identical to the *K. pneumoniae* serotype O1 chains of the LPS. More interestingly, the EPOL from KpMn7 is an ionic polymer rich in rhamnose monosaccharides, which may confer a less polar character to the backbone for the presence of methyl groups on their carbon 6 position instead of the more common primary alcohol function.

Considering the latter EPOL, its interaction with both BMAP-27 and Bac7(1–35) AMPs resulted in its being the lowest of the three polysaccharides, as determined by CD analysis and MIC assays. This behaviour can be traced back to the less-polar character of the polysaccharide backbone, which might produce three-dimensional conformations not able to establish a stable and strong interaction with the peptides. In fact, the intrinsic propensity of a polysaccharide to adopt an elongated chain conformation or a more compact and folded one, may strongly affect its capacity to interact with the peptide in the α-helix conformation. On one hand, the helical conformation may favour the interaction with the polysaccharide backbone due to its amphiphilic nature, on the second hand its rod-like nature could require an extended conformation of the polysaccharide for a stable interaction. The other two investigated polysaccharides reduce the antimicrobial activity of both peptides, offering a stronger protection to bacteria against these molecules. However, they behave differently with respect to BMAP-27 and Bac7(1–35). In the presence of the former, the interaction, and the consequent protection, is high in spite of their quite different ionic nature. Evidently, in this system ionic interactions are not essential to form suitable complexes between peptide and polysaccharides: different forces such as hydrogen bonds and van der Waals interactions may lead to an efficient complexation, causing conformational changes in the peptide. Bac7(1–35) showed a different scenario, where KpTs113 EPOL was the most efficient in inhibiting its antimicrobial action while the inhibitory effect of KpTs101 on peptide activity was intermediate between KpTs113 and KpMn7 EPOLs. Again, details of the chemical structure of the polysaccharides play a critical role in defining their biological action.

Considering together the spectroscopic evidence and results from microbiological assays, the interaction of both peptides with the biofilm polysaccharides leads to the formation of complexes, to some extent preventing the antimicrobials to reach the bacterial membrane or impeding a proper interaction with it, therefore lowering the activity of the peptide.

As a concluding remark, it can be speculated that the ability of bacteria belonging to the same species to biosynthesize EPOLs with very different primary structures is an efficient way to respond to external stimuli and threats. The chemistry of the EPOLs is then modulating the function of biofilms, which should not be considered as a passive scaffold enveloping bacterial colonies but an active tool in the hands of bacteria.

## Figures and Tables

**Table 1:** Primary structure of the exopolysaccharides extracted from biofilms of three *Klebsiella pnemoniae* strains—KpTs101, KpTs113, and KpMn7—and antimicrobial peptides aminoacid sequences.

*K. pneumoniae* Strain	Biofilm Polysaccharide Structure	Reference
KpTs101	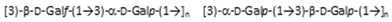	[14]
KpTs113	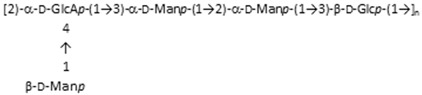	[15]
KpMn7	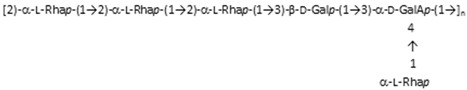	[16]
**Antimicrobial Peptide**	**Aminoacid Sequence**	
BMAP-27	GRFKRFRKKFKKLFKKLSPVIPLLHL-NH_2_	
Bac7(1-35)	RRIRPRPPRLPRPRPRPLPFPRPGPRPIPRPLPFP-NH_2_	

**Table 2:** Minimum inhibitory concentration (MIC) values of BMAP-27 and Bac7(1–35) against the three *Klebsiella* strains and the *E. coli* ML-35 strain used to evaluate the protective effect of the exopolysaccharides.

Bacterial Strain	BMAP-27	Bac7(1–35)
KpMn7	4 µM	4 µM
KpTs101	4 µM	4 µM
KpTs113	4 µM	4 µM
*E. coli* ML-35	2 µM	2 µM

## Abbreviations

AMPs	Antimicrobial Peptides
MH	Müller Hinton solid medium
EPOLs	Extracellular Polysaccharides
MIC	Minimum Inhibitory Concentration
CD	Circular Dichroism
